# The Predictive Validity of the Full Outline of UnResponsiveness Score Compared to the Glasgow Coma Scale in the Intensive Care Unit: A Systematic Review

**DOI:** 10.1007/s12028-024-02150-8

**Published:** 2024-11-05

**Authors:** Jaime E. Schey, Monica Schoch, Debra Kerr

**Affiliations:** 1 School of Nursing and Midwifery, Faculty of Health, Deakin University, Geelong, VIC Australia; 2 Intensive Care Unit, Royal Melbourne Hospital, Melbourne, VIC Australia; 3 Centre for Quality and Patient Safety Research, School of Nursing and Midwifery, Institute for Health Transformation, Deakin University, Geelong, VIC Australia; 4 Deakin University, Western Health Partnership, St Albans, VIC, Australia

**Keywords:** Coma, Critical care, Critical illness, Glasgow coma scale, Neurologic examination, Neurological manifestations, Prognosis

## Abstract

The Full Outline of UnResponsiveness (FOUR) score was developed to overcome the limitations of the Glasgow Coma Scale (GCS) when assessing individuals with impaired consciousness. We sought to review the evidence regarding the predictive validity of the GCS and FOUR score in intensive care unit (ICU) settings. This review was prospectively registered in PROSPERO (CRD42023420528). Systematic searches of CINAHL, MEDLINE, and Embase were undertaken. Prospective observational studies were included if both GCS and FOUR score were assessed in adults during ICU admission and if mortality and/or validated functional outcome measure scores were collected. Studies were excluded if they exclusively investigated patients with traumatic brain injury. Screening, data extraction, and quality assessment using the Quality in Prognosis Studies tool were conducted by two reviewers. Twenty studies of poor to moderate quality were included. Many studies only included patients with neurological illness and excluded sedated patients, despite high proportions of intubated patients. The FOUR score achieved higher area under the receiver operating characteristic curve values for mortality prediction compared with the GCS, and the FOUR score achieved significantly higher area under the receiver operating characteristic curve values for predictions of ICU mortality. Both coma scales showed similar accuracy in predicting “unfavorable” functional outcome. The FOUR score appeared to be more responsive than the GCS in the ICU, as most patients with a GCS score of 3 obtained FOUR scores between 1 and 8 due to preserved brainstem function. The FOUR score may be superior to the GCS for predicting mortality in ICU settings. Further adequately powered studies with clear, reliable methods for assessment of index and outcome scores are required to clarify the predictive performance of both coma scales in ICUs. Inclusion of sedated patients may improve generalizability of findings in general ICU populations.

**Supplementary Information:**

The online version contains supplementary material available at 10.1007/s12028-024-02150-8.

## Introduction

Neurological assessment findings inform decisions regarding the initiation, escalation, and withdrawal of life-supportive therapies in the intensive care unit (ICU) [1, 2]. However, consciousness and neurological function are subjective constructs and thus rely on skillfull assessment by individual clinicians [1–3]. Subsequently, the use of a clinical assessment tool for neurological assessment remains a best practice recommendation, as such tools optimize consistency and communication among clinicians [4]. The Glasgow Coma Scale (GCS) remains the gold standard for serial neurological assessments across acute settings, including ICUs. However, the GCS has several limitations when applied in ICU settings: all GCS components are readily confounded by sedation [5], the verbal component is not assessable in intubated patients [6, 7], brainstem reflexes are not assessed [6, 7], and responsiveness is poor when assessing patients with low levels of consciousness [6–8]. A novel alterative, the Full Outline of UnResponsiveness (FOUR) score, was developed to overcome these shortcomings [6, 7, 9, 10].

**Fig. 1:**
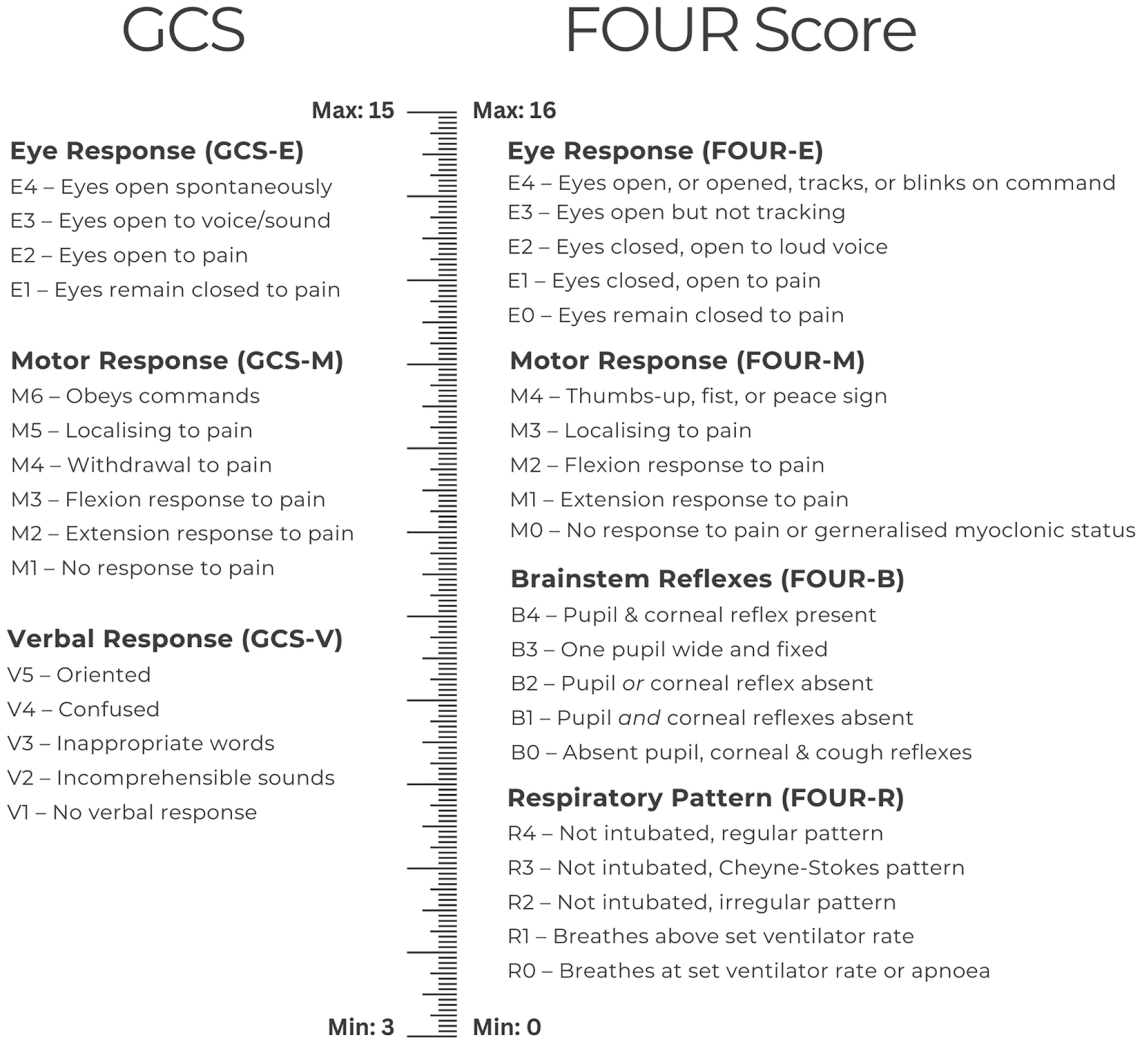
A comparison of the Glasgow Coma Scale (GCS) and the Full Outline of UnResponsiveness (FOUR) Score. Adapted from Wijdicks et al. [7]

The GCS consists of three components: eye (GCS-E), verbal (GCS-V), and motor (GCS-M) [11]. In contrast, the FOUR score consists of four components: eye (FOUR-E), motor (FOUR-M), brainstem (FOUR-B), and respiratory pattern (FOUR-R; as shown in Fig. 1). Unlike the GCS, all FOUR score components can be assessed in most unconscious and intubated ICU patients [2, 6, 7]. The eye and motor components of the two tools are similar, although the FOUR score is more detailed [4, 6, 12]. To achieve the highest FOUR-E score, a patient must demonstrate visual pursuit [7], which indicates a degree of cortical functioning in seemingly unconscious patients [3, 13, 14]. The FOUR motor lists myoclonic status alongside no response (to painful stimuli) as the lowest possible score [7]. Unlike the GCS, the FOUR score includes assessment of brainstem reflexes (FOUR-B) [15]. Lower brainstem function is assessed through the (FOUR-R) component, which assesses respiratory effort above the ventilator rate for intubated patients [7].

### Rationale and Objective

Despite multiple theoretical advantages, the FOUR score is yet to be widely adopted into clinical practice and research [6, 10, 16]. The authors of the FOUR score affirm the tool’s mathematical precision, which allows the use of the sum score as an independent measure of disease severity [7, 10, 17]. Conversely, the authors of the GCS do not recommend the use of the GCS sum score alone as a prognostic factor, or measure of disease severity [1, 18], as the association between GCS and patient outcomes remains controversial [6, 18–20]. Although neither the GCS nor the FOUR score was intended for use in outcome prediction, an evaluation and comparison of predictive validity (a type of criterion validity) is needed to provide further insight into each tool’s psychometric validity and clinical utility in ICU settings [21]. Existing reviews have evaluated the predictive performance of the FOUR score in acute settings and in patients in ICUs with traumatic brain injury (TBI) [9, 10, 12, 17]. A recent review by Brun et al. [22] found the FOUR score had higher interrater reliability, internal consistency, and content validity compared with the GCS in critical care settings. Furthermore, the European Society of Intensive Care Medicine 2014 expert panel report on neurological assessment of the ICU patient recommends the use of the FOUR score for further differentiation of low GCS scores but noted the limited evidence comparing the predictive value of the two tools [21]. However, to our knowledge, no existing review has compared the predictive performance of the FOUR score with the GCS in ICU settings. Therefore, we aimed to systematically review evidence regarding the predictive validity of the FOUR score compared to the GCS in predicting mortality and functional outcome measure (FOM) scores for adults hospitalized in ICU settings.

## Methods

The protocol for this systematic review was developed in line with the Cochrane Handbook for Systematic Reviews of Diagnostic Test Accuracy [23], and was prospectively registered in PROSPERO (CRD42023420528) before searches began. This review is reported according to the 2020 Preferred Reporting Items for Systematic Reviews and Meta-Analyses (PRISMA) statement [24].

### Eligibility Criteria

Prospective observational studies were eligible for inclusion if all items of both the GCS and FOUR score (index measures) were used to assess adults (≥ 16 years) in an ICU setting. Eligible studies were also required to have measured the index measures’ association with a primary outcome measure: mortality or scores of a validated FOM tool (e.g., Glasgow Outcome Score [GOS]/GOS Extended [GOSE]; Modified Rankin Scale [mRS]; Cerebral Performance Category [CPC]). Other predictive criterion measures (e.g., extubation failure) encountered were included as secondary outcome measures. Only peer-reviewed articles with English full-text were eligible. Articles were included if study criteria were met, regardless of the quality assessment. Retrospective designs, case series and conference abstracts were excluded. Studies exclusively investigating TBI cohorts were also ultimately excluded, as the predictive performance of the GCS and FOUR score in this distinct subpopulation has been well documented in numerous reviews [9, 10, 17], including a recent systematic review and meta-analysis by Ahmadi et al. [12], and our review sought evidence relevant to general ICU cohorts. Studies reporting data from ICU and non-ICU samples were only included if ICU patient data were reported separately.

### Information Sources and Search Strategy

Systematic searches of MEDLINE, Embase and CINAHL were limited to articles published from 2005 (when the FOUR score was introduced) [7] until the main search concluded in July 2023. No other filters or limiters were used. Full line-by-line search strategies were reviewed by a specialist librarian (Supplementary File 1). The search concluded following a manual search of the reference lists of included studies and similar systematic reviews [9, 10, 12, 17]. Searches were repeated in July 2024 to ensure no eligible studies from the preceding 12 months were missed.

### Study Selection and Data Extraction

Citations were exported to EndNote (version 21.2). Once all searches were conducted, references were imported to Covidence (www.covidence.org) for deduplication and screening. Titles and abstracts of remaining references were independently screened by two authors for potential inclusion, and full-text review. Two authors then independently assessed full texts of each remaining article against selection criteria. Corresponding authors were contacted directly if further clarification was required, or if we were unable to source manuscripts in English. Two reviewers independently extracted data using a customized form in Covidence. Data items included methodological and clinical characteristics, and statistical data quantifying the index measures’ association with predictive outcome measures. Any disagreements at any point of the selection and extraction processes were resolved with further discussion, or input from a third author when required.

### Quality Assessment

Two reviewers used the Quality in Prognosis Studies (QUIPS) tool [25] to independently assess the Risk of Bias (RoB). The QUIPS tool consists of six domains that are each rated as low, moderate, or high RoB. Overall RoB for each study was determined with a ‘highest score counts’ approach, as recommended by the authors of the QUIPS tool [25]. Any disagreement was resolved with further discussion.

### Effect Measures and Data Synthesis

Study characteristics are presented in tables: methodological characteristics are shown in Table 2, and a summary of patient characteristics is provided separately in Supplementary File 3. Results regarding the predictive performance of the index measures are grouped by criterion measure (i.e., mortality or functional outcome) and the statistical measure quantifying predictive performance; namely, area under the receiver operating characteristic curve (AUROC), sensitivity and specificity, and/or unadjusted diagnostic odds ratios (ORs).

The AUROC statistic provides an average value of sensitivity for all specificity thresholds (and vice versa), and thus represents the overall diagnostic accuracy of a test [26]. A test with perfect accuracy has an AUROC = 1.0, while an AUROC = 0.50 indicates discriminatory abilities equal to chance [26]. Collected AUROC values were interpreted according to the recommendations by Hosmer et al. [27]: AUROC < 0.70 is considered inadequate, AUROC ≥ 0.70 indicates good or adequate accuracy, AUROC ≥ 0.80 indicates excellent accuracy, and AUROCs ≥ 0.90 indicates outstanding accuracy. Pooling of AUROC values through random-effects meta-analysis was considered, and the extent of heterogeneity was measured with the *I*^2^ statistic. The *I*^2^ statistic describes the percentage of effect estimate variance due to between-study heterogeneity (generally due to clinical or methodological differences) and not attributable to chance (i.e., sampling error). [27] However, substantial statistical heterogeneity (FOUR *I*^2^ = 71%; GCS *I*^2^ = 70%) was identified, which ultimately precluded meaningful meta-analysis.

## Results

### Search Results and Selection

**Fig. 2:**
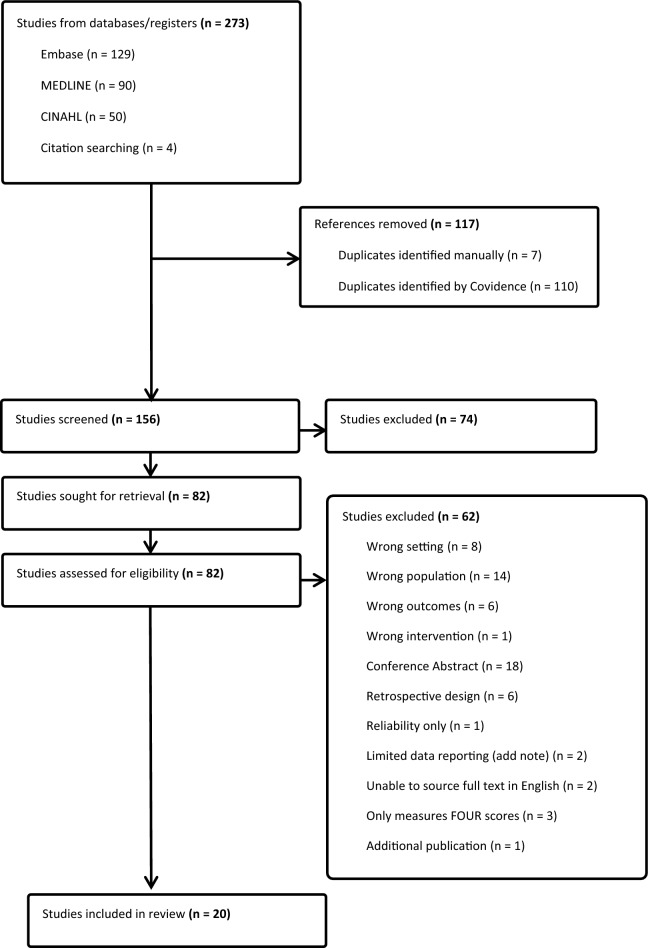
PRISMA flow diagram. CINAHL – Cumulative Index to Nursing and Allied Health Literature; PRISMA—Preferred Reporting Items for Systematic Reviews and Meta-Analyses

Following the search, 273 references were imported to Covidence for screening. After deduplication, the titles and abstracts of 156 articles were reviewed and 82 articles proceeded to full-text review. Sixty-two articles were ineligible and one article that met selection criteria was excluded [28], as this article described further analysis of previously reported data that were already included [30]. Full texts were unable to be sourced for two articles despite attempts to contact the authors directly. A total of 20 studies were included for review (Fig. 2) [7, 8, 30–47].

### Study Characteristics

The 20 prospective observational studies were conducted in either general ICUs (*n* = 11; 55%) [8, 30, 32–34, 36, 37, 42–45] or neuroscience ICUs (*n* = 9; 45%) [7, 31, 35, 38–41, 46, 47] where patients were recruited consecutively on ICU admission. There were 3,965 participants across the 20 studies, and sample sizes were generally small, ranging from 55 [36] to 1,645 [45]. The study with the largest sample (*N* = 1,645) was the only study to conduct a priori power analysis [45]. The remaining studies had samples of 300 patients or less, seven of which reported samples with less than 100 patients [34, 36, 38, 39, 42, 44, 46]. Three studies only included patients with specific neurological illnesses: stroke of any etiology [35], ischemic stroke [37], and aneurysmal subarachnoid hemorrhage [38]. Another four studies included patients with any acute neurological condition [34, 39, 41, 47], and three studies only included patients with low GCS (≤ 9) [8, 31, 42]. Nine studies excluded patients who were sedated [7, 8, 33, 35, 40, 41, 43, 46, 47], and two studies only included patients after cardiac arrest [32, 44]. Most studies assessed GCS and FOUR score at least once within 24 h of admission [9, 27, 28, 30–36, 38–44], as shown in Table 1. Five studies assessed FOUR and GCS scores on multiple occasions at various time intervals [32, 35–37, 44].

**Table 1:** Study Characteristics

StudyStudy	Country	ICU Setting	Selection Criteria		FOUR/GCS Assessment			Outcome Assessment			
			Inclusion	Exclusion	Assessor	Timing	Blinding	Measure	Timing	FOM Assessor	Blinding
Bruno 2011	Belgium	General	GCS < 8	S; NMBA	MD; RN; Npsy	≤ 1m^a^	Y	GOS	3 m	Inv	U
Chen 2013	Germany	Neurosciences	> 17yrs; GCS < 9	–	MD	≤ 24hrs^a^	U	† GOS	30d	MD	U
Fugate 2010	USA	General	Post-arrest	–	MD	1-2d, 3-5d	N	† CPC	Dc	Inv	U
Iyer 2009	USA	General	ICU Adm	S; NMBA	MD; RN	n.s	U	† mRS	Dc 3 m	Inv	U
Khanal 2016	Nepal	General	> 16yrs; Neuro.ill; ≥ 24 h ICU	–	Inv	≤ 24hrs^a^	U	†	Dc	–	–
Kocak 2012	Turkey	Neurosciences	Stroke	S; NMBA	MD	0 h, 1d, 3d & 10d	U	†	≤ 15d	–	–
Kwamboka 2022	Kenya	General	> 16yrs; ≤ 48 h in ICU	–	RN	0d; 14d	N	†	14d	–	–
Mansour 2015	Egypt	General	> 18yrs; Isch.stroke	S^; NMBA	Inv	24hrs^a^, 72hrs^a^	U	† mRS	Dc & ≤ 30d 3 m	Inv	U
Mishra 2019	India	Neurosciences	aSAH	S^; PsycHx; non-aSAH	MD	≤ 24hrs^a^	U	† GOS	28d	MD	U
Örken 2010	Turkey	Neurosciences	ICU Adm	S; NMBA	MD	–	U	† mRS	Dc Dc, 30d	U	U
Olsen 2020	Denmark	Neurosciences	Neuro.ill	–	RN	–	U	GOSE	6 m	U	U
Peng 2015	China	Neurosciences	≥ 18yrs; TBI or Neuro.ill	S; NMBA; ˇBP	MD; RN	< 1d	U	†mRS	Dc 3 m	U	U
Ramazani 2019	Iran	General	ICU Adm	B.Death; ≥ 24 h in ICU	Inv	≤ 24hrs^a^	U	†	Dc	–	–
Said 2016	Bahrain	General	GCS ≤ 8; Mech.V	S^; NMBA; Trache	MD	≤ 24hrs^a^	U	†mRS Extubation	30d 3 m 14d	Inv	U
Suresh 2019	India	General	≥ 18yrs	S; NMBA	MD; RN	–	U	†GOS mRS	Dc	Inv	U
Weiss 2015	France	General	OHCA; GCS ≤ 8	Cog.Imp; GOC	MD	1d-7d	N	†CPC	6 m	Inv	U
Wijdicks 2005	USA	Neurosciences + referrals	ICU Adm	S; NMBA	MD; RN	1d	U	†mRS	Dc3m	Inv	U
Wijdicks 2015	USA	General	APACHE IV ICU	–	MD; RN	< 1hr^a^	U	†	Dc	–	–
Wolf 2007	USA	Neurosciences + referrals	ICU adm	S	RN	≤ 24 h	Y	†mRS	Dc30d	Inv	U
Zhao 2021	China	Neurosciences	GCS ≤ 7	S; ≥ 72 h in ICU	MD	Adm	N	†mRS	Dc3m	MD	Y

Neurosciences ICUs refer to those caring for those with neurosurgical or neurocritical illnesses exclusively. Studies that did not explicitly state the type of ICU were presumed to be a general or ‘mixed’ ICU

Selection Criteria: *Adm* – Admission; *APACHE IV ICU –* Admission to ICU using APACHE IV database. *B.Death* – Brain death; ˇBP – hypotension; *Chronic.ill* – Chronic or terminal illness; *Cog.Imp* – Cognitive impairment; *GOC* – Limitations on goals of (ICU/resuscitative) care; *Isch.stroke* – Ischaemic stroke; *Mech.V* – Mechanically ventilated; *Neuro.ill* – Neurological illness; *Neur.deg./Neur.musc.* – Neurodegenerative or neuromuscular diseases; *NMBAs* – exposure to Neuromuscular Blocking (paralytic) Agents; *PsycHx* – history of psychiatric illness; *S* – Sedated/exposure to sedatives; *S^* – Sedated heavily (some sedation allowed)*; SCI* – Spinal Chord Injury; *Sensˇ* – Sensory deficits; *aSAH* – Aneurysmal Subarachnoid Haemorrhage; *Trache* – Tracheostomy; *TBI* – Traumatic Brain Injury

Assessor: *Inv* – Investigator of unspecified craft group; *MD* – Doctor; *NPsy* – Neuropsycholgist; *RN* – Nurse

Timing: Time of index/FOM assessment; *Adm* – upon admission; *Dc* – upon discharge; Or hours (hrs), days (d), weeks (wks) or months (m) from admission

Blinding: U – unclear if assessors of index or FOM were blinded to patient data, history, or other predictive factors; *N* – Reason to believe index or FOM assessors were not blinded to patient data, history or other predictive factors; *Y* – Evidence that index or FOM assessors were blinded to patient data, history and other predictive factors

Outcome Measure: † – mortality; *CPC –* Cerebral Performance Category; *GOS(/E)* – Glasgow Outcome Scale(/Extended); *mRS* – Modified Rankin Scale

### Quality Assessment Findings

Quality assessment using the QUIPS tool [25] found 12 studies (60%) to have moderate overall RoB [7, 8, 31, 33, 37, 38, 41, 42, 44–47] and eight studies (40%) had high RoB [30, 32, 34–36, 39, 40, 43]. Risk of bias due to confounding (QUIPS Domain 5) was the most common reason for higher RoB scores, primarily due to a lack of adjustment for other prognostic factors. Methods for index and outcome measure assessment and data collection (QUIPS Domains 3 and 4) were often poorly described, therefore, it was difficult to ascertain how reliability was optimized. A traffic light plot and summary plot of RoB assessment findings are provided in Supplementary File 2.

### Patient Characteristics

Mean age ranged from 40.1 [43] to 70.5 [35] years, and there was an even distribution of sex (51% male, *n* = 2020; 49% female, *n* = 1965). Neurological diseases with nontraumatic etiologies were the most common causes for ICU admission (*n* = 1,404). Among these, the most common primary diagnoses included ischemic stroke (*n* = 403) and nontraumatic intraparenchymal hemorrhage or subarachnoid hemorrhage (*n* = 356). Five percent of the total sample had a TBI (*n* = 195). Common nonneurological causes for ICU admission included cardiopulmonary arrest (*n* = 321) and sepsis (*n* = 48). Four studies reported the proportion of sedated patients [31, 36, 44, 45], which ranged from 16.5% [45] to 91% [31], and approximately 46.5% of the overall sample were intubated. Patient characteristics are summarized in Supplementary File 3.

#### Distribution of Index Scores

Mean index sum scores reported in five studies ranged from 2.4 [44] to 13.6 [38] for the FOUR score, and 3.5 [44] to 12.6 [38] for the GCS [30, 31, 34, 38, 44]. Median index scores reported in four studies [37, 39, 42, 47] ranged from 7 [39, 47] to 11 [37] for the FOUR score, and 5 [47] to 8 [37] for the GCS [6, 38, 41, 46]. A summary of mean and median index measure scores is provided in Supplementary File 4.

### Mortality

Eighteen studies measured mortality as a primary outcome [7, 30–38, 40–47], of which 11 studies measured in-hospital mortality [7, 30, 32–34, 37, 40, 41, 43, 45, 46], two studies measured in-ICU mortality [45, 47], and six studies evaluated mortality within 30 days of ICU admission [31, 35–38, 42]. One study investigated mortality at 3 months [47] and another study measured mortality at 6 months [44]. Mortality rate was generally high but varied, ranging from 12% [45] to 70% [35].

#### Area Under the Curve

Fourteen studies reported AUROC values to quantify the accuracy of the FOUR score and GCS in predicting mortality at various time points [7, 30, 31, 33–38, 41, 42, 44, 45, 47]. Seven studies (two with high RoB [30, 34] and five with moderate RoB [7, 33, 37, 41, 45]) collected index scores within 24 h of ICU admission, and reported AUROCs for hospital mortality prediction, as shown in Table 2. The FOUR score achieved excellent AUROC values (AUROC ≥ 0.80) for predictions of hospital mortality in six studies [7, 30, 33, 34, 37, 41], and one study reported an AUROC indicative of adequate accuracy (AUROC = 0.70; 95% CI 0.66–0.74) [45]. FOUR scores collected within 24 h of admission achieved higher AUROC values than the GCS for hospital mortality prediction in all but one of the seven studies [30, 33, 34, 37, 41, 45, 47], which reported equivalent AUROCs for both index measures [7]. Five studies reported excellent AUROC values for the GCS [7, 30, 33, 41]. Two studies found the GCS to be adequate (AUROC ≥ 0.70) for prediction of hospital mortality [34, 37]. In contrast, Wijdicks et al. [45] reported GCS AUROC values indicative of inadequate predictive performance regarding hospital mortality (AUROC = 0.68; Table 2).

**Table 2:** Summary of AUROC Values for Prediction of Mortality at Various Timepoints

Study	Mortality Timepoint	Index Assessment Timing	FOUR		GCS	
			AUROC	95% CI	AUROC	95% CI
Iyer 2009	Hospital Dc	–	0.86	–	0.82	–
Khanal 2016	Hospital Dc	≤ 24 h	0.82	0.73–0.91	0.79	0.74–0.90
Mansour 2014	Hospital Dc	24 h 72 h	0.80 0.98	0.72–0.86 0.93–1.0	0.78 0.98	0.68–0.85 0.93–0.94
Peng 2015	Hospital Dc	≤ 24 h	0.83	0.74–0.93	0.82	0.72–0.91
Ramazani 2019	Hospital Dc	≤ 24 h	0.87	0.83–0.91	0.83	0.78–0.87
Wijdicks 2005	Hospital Dc	≤ 24 h	0.81	–	0.81	–
Wijdicks 2015	Hospital Dc ICU Dc	≤ 1 h	0.74 0.70	0.69–0.79 0.66–0.74	0.72* 0.68	0.66–0.77 0.64–0.72
Zhao 2021	ICU Dc	24 h 72 h	**0.76** 0.84	**0.71–0.81** 0.79–0.88	**0.64** 0.77	**0.57–0.69** 0.72–0.82
Kocak 2012	≤ 15 days	≤ 24 h 3d 10d	0.68 0.92 0.98	0.57–0.79 0.87–0.98 0.95–1.0	0.62 0.93 0.98	0.51–0.74 0.87–0.98 0.95–1.0
Kwamboka 2022	14 days	≤ 24 h	0.76	0.44–0.83	0.63	0.47–0.85
Chen 2013	30 days	≤ 24 h	0.77	0.66–0.87	0.70	0.60–0.80
Mishra 2019	28 days	≤ 24 h	0.80	0.68–0.91	0.78	0.67–0.90
Said 2016	30 days	≤ 24 h	0.86	0.79–0.94	0.84	0.75–0.93

Index assessment timing refers to time from ICU admission

Dc – discharge

^*^p < 0.001 Bold numbers highlight confidence intervals that do not overlap

Two studies [45, 47] with moderate RoB, reported AUROC values for GCS and FOUR scores’ (collected within 24 h of admission) predictions of ICU mortality [45, 47]. Both studies [45, 47] found the FOUR score achieved significantly higher AUROC values for ICU mortality prediction compared to the GCS, as shown in Table 2.

Regarding mortality within 15 or 30 days, the AUROC values for the GCS and FOUR score varied (Table 2). Both studies [35, 36] assessing predictions of mortality within 15 days were of high RoB. Kocak et al. [35] collected both FOUR and GCS scores on day 0 of ICU admission and found both index measures to be inadequate for 15-day mortality prediction (AUROC = 0.68 for FOUR score; AUROC = 0.62 for GCS), however, reported considerably improved AUROCs (indicating outstanding accuracy) for both scores when assessed on day 3 and day 10 of ICU admission. Kwamboka and Kerubo [36] collected index scores within 24 h of admission and found the AUROC for the GCS to be inadequate (AUROC = 0.63) in predicting 14-day mortality, although the AUROC for the FOUR score was sufficient (AUROC = 0.76).

Only two studies investigated longer-term mortality (≥ 3 months) [44, 47]. Weiss et al. [44] compared AUROCs representing the predictive value of nonimprovement in index sum scores between days 1 and 3 for predictions of mortality at 6 months. They found that a lack of improvement in FOUR score by at least 1-point over the three days was more accurate in predicting mortality at 6 months (AUROC = 0.84), compared to lack of improvement in GCS (AUROC = 0.75). Zhao et al. [47] collected GCS and FOUR scores within 24 h of admission and found both measures were inadequate in predicting 3 month mortality, with GCS AUROC values indicating particularly poor discriminative abilities (AUROC 0.52; 95% CI 0.46–0.58). The AUROC values for the FOUR score were higher (AUROC 0.63; 95% CI 0.57–0.69), but also inadequate for prediction of 3-month mortality.

#### Sensitivity and Specificity

Ten studies reported sensitivity and specificity of the index measures’ predictions of mortality at various time points (ST2 in Supplementary File 4) [7, 30–32, 34, 37, 38, 41, 42, 48]. Neither index measure appeared to be more sensitive for mortality prediction at any time point, although variation in cut off values limits direct comparison. However, seven of the ten studies found the FOUR score had higher specificity compared to the GCS [30–32, 34, 38, 42, 44], including all three studies of moderate RoB that measured 30-day mortality [31, 38, 42].

#### ORs and Akaike Information Criterion

Seven studies estimated the odds of mortality at hospital discharge [7, 33, 34, 37, 45, 46], or within 30 days [37], as shown in Supplementary File 4 (ST3). All studies found a significant association between sum scores and mortality for both index measures. ORs for hospital mortality and FOUR/GCS score (1-point increase) ranged from 0.62 [46] to 0.80 [7] for the FOUR score, and 0.21 [46] to 0.74 [7] for the GCS.

Wijdicks et al. [45] calculated ORs for a 25% decrease in each sum score collected within 24 h of admission and found that lower GCS or FOUR score significantly increased the odds of both in-ICU mortality and in-hospital mortality [45]. Odds ratios were higher for the FOUR score (OR = 2.72; OR = 2.76 for hospital and ICU mortality, respectively) compared to the GCS (OR = 2.00; OR = 2.04 for hospital and ICU mortality, respectively) [45]. Another study [43] reported Akaike’s information criterion (AIC) values, a method for comparing the relative goodness-of-fit of regression models, that is, which model minimizes information loss without increasing model complexity, where the lower AIC indicates the better-fitting model (and implies a lower possibility of prediction error). This study found the AIC value for the FOUR score (AIC 105.46) indicated a better-fitted model compared to the GCS (AIC 109.47) for prediction of in-ICU mortality, although index measure assessment timing was unclear.

#### Association Between Lowest Index Scores and Mortality

Ten studies reported the number of patients with the lowest GCS score (GCS 3; n = 335) [7, 8, 31–33, 39, 40, 44–46]. However, only 127 of these patients also scored the lowest possible FOUR score (FOUR 0; ST4, shown in Supplementary File 4). Patients with a GCS of 3 were assessed to achieve FOUR scores up to 8, which indicates preserved brainstem functioning. Additionally, four studies reported outcomes for patients with the lowest FOUR/GCS scores (ST5 in Supplementary File 4) [8, 32, 33, 46]. Fifteen percent (n = 16) of the 105 patients with a GCS of 3 survived, whereas only one (1.8%) of the 55 patients with a FOUR score of 0 survived.

### Functional Outcome

Fifteen studies investigated the performance of the GCS and FOUR score in predicting functional outcome [7, 8, 31–33, 37–44, 46, 47]. Functional outcome assessments occurred either between 3 and 6 months [7, 8, 33, 37, 39, 41, 42, 44, 47], within 30 days of admission [31, 38, 40, 46], or upon hospital discharge [32, 40, 43]. Most studies (*n* = 9) assessed mRS [7, 33, 37, 40–43, 46, 47], four measured GOS [8, 31, 38, 43], one assessed GOSE [39], and two assessed CPC [32, 44]. Ordinal FOM scores were dichotomized to represent either “favorable” or “unfavorable” outcomes, with consistent cut off thresholds: an mRS of 3–6 or a GOS of 1–3 indicated unfavorable outcomes. Death (a mRS of 6 or a GOS of 1) was included as a potential unfavorable outcome in all studies, except for Chen et al. [31] who excluded patients who had died (GOS 1) from their FOM prediction analysis.

#### Area Under the Curve

Nine studies reported AUROC values for prediction of unfavorable FOM scores that were assessed either within 30 days of admission [31, 38], or at 3 months [7, 8, 33, 37, 41, 42, 47], as shown in Table 3. The AUROC values for prediction of ‘unfavorable’ FOM scores at 3 months using GCS and FOUR scores appeared equivalent and indicative of adequate accuracy in most studies, with values ranging from AUROC 0.66 [47] to 0.91 [37] for the FOUR score, and from 0.55 [47] to 0.93 [37] for the GCS. Zhao et al. [47] found both index measures (when assessed within 24 h) were inadequate in predicting 3-month mRS, although the FOUR score achieved a much higher AUROC value (AUROC = 0.66) compared to the GCS (AUROC = 0.55). Bruno et al. [8] also found the GCS to be an inadequate predictor of 3-month unfavorable GOS scores (AUROC = 0.68), and the FOUR score achieved only slightly higher AUROC values (AUROC = 0.70; indicating adequate predictive performance). However, the particularly wide index score assessment window (within 1 month of admission; median = 8 days since ICU admission) should be noted, as this extended time frame will almost certainly introduce high variability in index scores, which may account for the reduced precision reported in this study. Additionally, Weiss et al. [44] found a lack of improvement in the FOUR score between days 1 to 3 after cardiac arrest was far more accurate in predicting poor outcome (CPC 3–5) at 6 months (AUROC = 0.87; 95% CI 0.74–0.94) compared to a lack of improvement in GCS (AUROC = 0.75; 95% CI 0.56–0.86) Nevertheless, five other studies that included a FOM at 3 months found both scores achieved AUROC values representing adequate to outstanding accuracy [7, 33, 37, 41, 42].

**Table 3:** Summary of AUROC Values for the FOUR and GCS in Predicting FOM Scores

Study	FOM Tool & Timepoint	Index Assessment Timing	FOUR Score		GCS	
			AUROC	95% CI	AUROC	95% CI
Bruno 2011	GOS 1–3 3 m	≤ 1 month	0.70	–	0.68	–
Iyer 2009	mRS 3–6 3 m	–	0.75	–	0.76	–
Mansour 2014	mRS 3–6 3 m	24 h 72 h	0.87 0.91	0.79–0.92 0.84–0.95	0.87 0.93	0.80–0.92 0.87–0.97
Peng 2015	mRS 3–6 3 m	≤ 24 h	0.82	0.74–0.89	0.81	0.73–0.89
Said 2016	mRS 3–6 3 m	≤ 24 h	0.91	0.85–0.97	0.90	0.83–0.96
Wijdicks 2005	mRS 3–6 3 m	≤ 24 h	0.72	–	0.72	–
Zhao 2021	mRS 3–6 3 m	24 h 72 h	0.66 0.69	0.60–0.71 0.63–0.75	0.55 0.61	0.49–0.61 0.55–0.67
Chen 2013	GOS 2–3 30d GOS 4–5 30d	≤ 24 h	0.68 0.75	0.53–0.83 0.62–0.87	0.68 0.73	0.53–0.83 0.59–0.87
Mishra 2019	GOS 1–3 28d	≤ 24 h	0.81	0.72–0.90	0.81	0.72–0.90

Index assessment timing refers to time from ICU admission

GOS 1–3 or 2–3 – unfavourable functional outcome, GOS 4–5 – favourable functional outcome

mRS 3–6 – unfavourable functional outcome

#### ORs and Correlation

Five studies estimated odds of ‘unfavorable’ functional outcome for a cumulative 1-point increase in index measure sum score [7, 8, 33, 37, 46]. Four of these studies conducted FOM assessments at 3-months [7, 8, 33, 37], while Wolf et al. [46] collected mRS assessment data at 30 days after ICU admission. The reported odds of an ‘unfavorable’ outcome at 3-months varied for both index scores, ranging from 0.53 to 0.86 for the FOUR score, and 0.42 to 0.89 for the GCS (ST6 in Supplementary File 4).

Örken et al. [40] found the FOUR score had a significantly stronger positive correlation with mRS on day 30 or discharge (r = 0.60) compared with the GCS (r = 0.54; *p* = 0.01). Olsen et al. [39] reported 13 of the 36 patients (36%) with a GCS of 3 had a favorable outcome at 6 months (GOSE ≥ 5), while all seven patients with a FOUR score of 3 or lower had an unfavorable outcome (GOSE ≤ 4).

### Secondary Outcomes

Only one study investigated a secondary outcome by including extubation failure at 14 days as a predictive criterion measure [42]. They found the FOUR score assessed within 24 h of admission was significantly more accurate (AUROC = 0.87; 95% CI 0.80–0.94) for prediction of 14-day extubation failure, compared to the GCS (AUROC = 0.83; 95% CI 0.74–0.92; *p* = 0.014).

## Discussion

To our knowledge, this is the first systematic review to compare the predictive validity of the FOUR score and GCS when applied in ICU settings. Our review of 20 studies found both the GCS and FOUR score to have significant associations with mortality and poor functional outcome for patients receiving care in ICUs. The type of ICU setting, sample sizes, selection criteria, and timing of outcome measurement varied for each precluded meta-analysis. However, most studies included patients with either neurocritical conditions or low levels of consciousness in the absence of sedation. Overall methodological quality was poor to moderate.

The findings of this systematic review suggest that the FOUR score may be superior to the GCS for predictions of mortality in ICU settings. The FOUR score achieved AUROC values indicating good to excellent accuracy in predicting ICU and hospital mortality, mortality within 30 days, and poor functional outcome. The predictive accuracy of the GCS appeared less consistent for mortality predictions. Both coma scales had comparable sensitivity in predicting mortality; however, the FOUR score appeared more specific. Notably, most studies did not find a significant difference in the accuracy of mortality predictions between the FOUR score and GCS [7, 30, 31, 34–38, 41, 42]. This is likely due to the included studies’ small samples (only one study [45] was adequately powered). However, these findings may also be distorted by additional, unmeasured predictive factors, as confounding (QUIPS Domain 5) due to lack of adjustment for other prognostic factors was the most common reason for higher RoB scores.

The FOUR score was shown to be significantly more accurate than the GCS for ICU mortality prediction in two of the higher quality studies with moderate RoB [45, 47]. Wijdicks et al. [45] (the only adequately powered study) enrolled a particularly large, diverse cohort of patients from 13 ICUs (*N* = 1,645), including patients receiving sedation and those with nonneurocritical conditions, however only 9% (*n* = 149) of their sample were intubated and sedated. While the difference in AUROCs reported by Wijdicks et al. [45] was significant, this difference was small (FOUR AUROC 0.742; GCS AUROC 0.715; *p* = 0.001). Zhao et al. [47] reported a relatively large difference between FOUR score and GCS AUROCs, however, their sample represented a distinctly different ICU cohort. Zhao et al. [47] recruited patients with neurosurgical conditions without sedation and their sample (*N* = 271) was considerably smaller than that reported by Wijdicks et al. [45]. Subsequently, the clinical significance of the statistically significant differences favoring the FOUR score remains unclear. However, these results complement the findings of Brun et al. [22], who found the FOUR score to be psychometrically superior to the GCS in critical care settings due to higher interrater reliability, internal consistency and content validity compared to the GCS in critical care settings.

The predictive performance was comparable for both index measures in predicting poor functional outcome, however, the FOM assessment tools and timepoints varied. Most studies assessed FOM at 3 months and two studies conducted FOM assessments within 30 days of admission [31, 38], which may be too early for ICU patients with neurological conditions [49, 50]. Only two studies [39, 44] measured functional outcome at 6 months, and both found the FOUR score to have a stronger relationship with poor outcome.

There was a considerable lack of clinical diversity in most included studies, which may limit the application of findings outside of ICU cohorts with large proportions of patients with neurological conditions or severely impaired consciousness in the absence of sedation. Many studies investigating the accuracy of the index measures in predicting mortality and functional outcome only included patients with neurological illness [7, 8, 31, 33, 34, 37, 38, 41, 42, 47], or they excluded sedated patients despite high proportions of intubated patients [7, 8, 33, 37, 41, 42, 47]. This is notable, as mechanical ventilation is generally not tolerated without sedation in the absence of neurological pathology or severe critical illness [51]. Only four studies reported varying proportions of sedated patients [31, 36, 44, 45], and it was unclear if sedated patients were assessed in another four studies [30, 32, 34, 39]. This lack of clinical diversity in current evidence was also identified in three previous reviews undertaken to investigate the predictive validity of the FOUR score [9, 10, 17].

Only one study [42] reported an additional predictive criterion measure, finding the FOUR score to be significantly more accurate in predicting extubation failure at 14 days compared to the GCS. However, this study was of moderate quality, as the patient cohort had additional prognostic factors that were not adequately described (e.g., predominant ventilator mode and weaning strategies). As such, further studies are required to substantiate these findings.

This review also found evidence suggesting the FOUR score may be more responsive than the GCS when used to assess comatose patients, as most patients with the lowest GCS (3) achieved FOUR scores above 0 due to preserved brainstem functions. Responsiveness describes the degree to which a tool can accurately detect clinically significant change [52], and a lack of responsiveness has been a well-known limitation of the GCS in the often sedated and intubated ICU patient [3, 6, 12, 15]. Unlike the GCS, the FOUR-B component can be assessed in patients receiving neuromuscular blocking agents and deep sedation, as pupillary light reflexes often remain intact [53]. Subsequently, the FOUR-B component, and therefore the overall FOUR sum score, may be less likely to be affected by sedation compared to the GCS. However, most included studies did not recruit patients who were receiving sedatives, despite high proportions of intubated patients. Therefore, the predictive value of the FOUR-B component may be underestimated in current evidence.

### Implications for Clinical Practice in the ICU

Our findings suggest that the FOUR score may be superior to the GCS for predictions of ICU mortality, which may have implications for existing and upcoming ICU mortality prediction models. Such models provide objective measures of critical illness severity and are essential for benchmarking and quality assurance monitoring in intensive care [54]. However, most existing models, such as APACHE [55–57] and ANZROD [58], currently include the lowest GCS score acquired within the first 24 h of admission and may exclude this variable if it is unable to be assessed due to sedation [58]. Substituting the GCS with a more accurate variable, such as the FOUR score, may further calibrate such models. Improving the calibration of ICU mortality prediction models may expand their use in clinical decision support and resource allocation, thereby further optimizing decision-making in the ICU [54, 59].

The potentially superior responsiveness of the FOUR score may assist in earlier detection of clinically relevant changes in intubated and sedated patients, and thus, expedite further assessment or intervention. Unlike the GCS, a FOUR sum score of 0 can alert clinicians to consider the clinical diagnosis of imminent brain death in the absence of confounders (e.g., high or toxic doses of barbiturates, baclofen or antidepressants, physiological extremis [60]) [3, 7]. Two retrospective observational studies [61, 62] found diagnosis of imminent brain death using the FOUR score alone to be a more accurate indicator of actual brain death compared to the GCS combined with absent brainstem reflexes. Furthermore, it has been suggested that use of the FOUR score in ICU settings may expedite recognition of potential beating-heart organ donors, thereby increasing the likelihood of donation [61, 62]. The FOUR score may also prompt clinicians to assess for locked-in syndrome, which is often mistaken for a vegetative state in approximately 50% of cases [63]. The preservation of vertical eye movement is a key feature of the syndrome [63, 64], which the FOUR score can identify through the assessment of visual pursuit with the FOUR-E component [14]. The European Academy of Neurology has also recommended the use of the FOUR score over the GCS to detect disorders of consciousness and emergence from such states [4].

Nevertheless, despite multiple strengths, the FOUR score has some weaknesses. Lack of a verbal component in the FOUR score may reduce responsiveness in mildly reduced conscious states, and subsequently, the FOUR score may be less useful outside of critical care settings [10]. The FOUR score requires knowledge of brainstem assessment and may be more difficult to apply. However, such assessment skills are within the scope of specialist ICU nurses and doctors, where high interrater reliability has been demonstrated [7, 33, 46, 65]. Additionally, Johnson and Whitcombe [66] found the face validity of the FOUR score to be far superior to the GCS among ICU nurses.

### Limitations

While this systematic review was conducted according to established international guidelines [23, 24, 67], several limitations are acknowledged. The search strategy was detailed but specific, and while it was reviewed by specialist librarians, some eligible articles may have been missed. Because non-English articles were excluded, our findings may be subject to language bias. Gray literature sources were not searched, as only peer-reviewed articles were sought to ensure our review summarized only high-quality evidence.

Our review found that included studies were likely underpowered, as only one study included a priori power analysis [45], and many studies reported samples of less than 100 patients [34, 36, 38, 39, 42, 44, 46]. As such, future research should involve power analysis to optimize the validity of any statistical effect estimate [68]. Furthermore, a considerable proportion of included studies were of poor methodological quality (40%). This was most commonly due to the potential influence of other predictive factors, which were generally unclear due to limited descriptions of baseline characteristics. Therefore, future studies should ensure detailed reporting of patient characteristics, and ideally, a measure of premorbid functioning and/or comorbidity severity. Including a validated disease severity score (such as APACHE, ANZROD, or SAPS) may assist in comparison of patient acuity and the external validity of findings. Additionally, most studies did not provide detailed descriptions of index and outcome measure assessment methods; therefore, it was unclear how reliability was optimized, and observer bias was reduced. Moreover, the validity of FOM scores may be further optimized if conducted at a longer interval, ideally at 6 to 12 months [49, 50].

Most studies only calculated or reported statistical analyses of index measure sum scores, rather than individual item scores. Reporting the accuracy for each component of both the GCS and FOUR score may further clarify which components are more useful, or perhaps redundant, in the ICU population. Finally, to further clarify the utility of the FOUR score in general ICU settings, sedated patients should not be excluded. As brainstem reflexes are often preserved even with deep sedation [53], the benefit of the FOUR score may become more evident if studied in cohorts with large proportions of intubated and sedated patients. Future reports may also describe the type and dose of sedatives, as this may assist in determining the degree in which different sedatives confound each component of the GCS and FOUR score.

## Conclusions

The FOUR score may be superior to the GCS for prediction of ICU and hospital mortality for ICU patients with low levels of consciousness. The FOUR score offers additional advantages over the GCS when used in the ICU setting, including increased responsiveness, which may improve efficiency in identifying and responding to deterioration. Consideration should be given to the inclusion of the FOUR score in ICU mortality prediction models to improve calibration. The FOUR score presents a promising alternative to the GCS in the ICU setting; however, this review’s findings are limited by a lack of clinical diversity. Although many studies assessed only patients with neurological illnesses, this review did not include samples with large proportions of patients with TBI, and few studies included patients receiving sedation. Therefore, broader conclusions regarding the general applicability of the FOUR score for all ICU patients, including those with TBI and/or those receiving sedation, cannot be drawn from this review alone.

Further studies with robust methodologies are required to clarify the psychometric and predictive performance of the FOUR score and GCS in ICU settings. Future studies should include patients with nonneurological illness and those receiving sedation, alongside detailed reporting of patient characteristics (including disease severity and comorbidities). As more evidence emerges, meta-regression analyses may clarify the extent to which sedation influences the predictive abilities of the GCS and FOUR score in ICU settings and facilitate comparisons of their prognostic performance across various ICU subpopulations.

## Supplementary Information

Below is the link to the electronic supplementary material.

Supplementary file1 (DOCX 26 kb)

Supplementary file2 (DOCX 597 kb)

Supplementary file3 (DOCX 23 kb)

Supplementary file4 (DOCX 30 kb)
