# Supplementary material for: The Predictive Validity of the Full Outline of UnResponsiveness Score Compared to the Glasgow Coma Scale in the Intensive Care Unit: A Systematic Review
Source: Neurocrit Care. 2024 Nov 5;43(2):645–58. doi: 10.1007/s12028-024-02150-8 (PMC12436514; doi:10.1007/s12028-024-02150-8)
Supplement: Supplementary file 1 — Supplementary file1 (DOCX 26 kb) [file 12028_2024_2150_MOESM1_ESM.docx]

**Supplementary Table 1. Medline Complete Search Strategy**

Note. Additional strategies available upon request.

| **#** | **Query** | **Results** |
| --- | --- | --- |
| S49 | S14 AND S17 AND S22 AND S47  Limiters - Date of Publication: 20050101-20231231 | 90 |
| S48 | S14 AND S17 AND S22 AND S47 | 94 |
| S47 | S46 OR ( (MH "Reproducibility of Results+") OR (MH "Sensitivity and Specificity+") OR (MH "Data Accuracy+") OR (MH "Investigative Techniques+") OR (MH “Predictive Value of Tests”) OR (MH "Prognosis+") OR (MH "Early Diagnosis+") OR (MH "Treatment Outcome+") OR (MH "Clinical Decision-Making+") OR (MH "Diagnosis+") OR (MH "Diagnostic Techniques and Procedures+") OR (MH "Diagnostic Errors+") OR (MH "Diagnosis, Computer-Assisted+") OR (MH "Outcome Assessment, Health Care+") OR (MH "Glasgow Outcome Scale") OR (MH "Outcome and Process Assessment, Health Care+") OR (MH "Treatment Outcome+") OR (MH "Patient Outcome Assessment+") OR (MH "Critical Care Outcomes") OR (MH "Simplified Acute Physiology Score") OR (MH "Adverse Outcome Pathways") OR (MH "Injury Severity Score") OR (MH “propensity score”) OR (MH "Mortality+") OR (MH "Hospital Mortality") ) | 20,349,055 |
| S46 | S23 OR S24 OR S25 OR S26 OR S27 OR S28 OR S29 OR S30 OR S31 OR S32 OR S33 OR S34 OR S35 OR S36 OR S37 OR S38 OR S39 OR S40 OR S41 OR S42 OR S43 OR S44 OR S45 | 6,179,868 |
| S45 | AB disagree* OR TI disagree* | 32,848 |
| S44 | AB agree* OR TI agree* | 462,512 |
| S43 | AB inter#observer OR TI inter#observer | 21,609 |
| S42 | AB inter#rater OR TI inter#rater | 10,909 |
| S41 | AB Reliabil* OR TI Reliabil* | 212,305 |
| S40 | AB mortality OR TI mortality | 976,435 |
| S39 | AB morbidity OR TI morbidity | 474,640 |
| S38 | AB (severity N3 score) OR TI (severity N3 score) | 40,429 |
| S37 | AB (Fisher Scale) OR TI (Fisher Scale) | 545 |
| S36 | AB (Hunt Hess) OR TI (Hunt Hess) | 1,847 |
| S35 | AB (Hunt-Hess) OR TI (Hunt-Hess) | 738 |
| S34 | AB (modified rankin scale) OR TI (modified rankin scale) | 13,815 |
| S33 | AB GOS OR TI GOS | 4,475 |
| S32 | AB (glasgow outcome score) OR TI (glasgow outcome score) | 2,737 |
| S31 | AB responsiveness OR TI responsiveness | 114,225 |
| S30 | AB clin?metric OR TI Clin?metric | 1,239 |
| S29 | AB Psychometric OR TI Psychometric | 57,796 |
| S28 | AB (construct N2 validity) OR TI (construct N2 validity) | 27,221 |
| S27 | AB (content N2 validity) OR TI (content N2 validity) | 13,454 |
| S26 | AB outcome* OR TI outcome* | 2,202,112 |
| S25 | AB prognos* OR TI prognos* | 792,689 |
| S24 | AB predict* OR TI predict* | 2,049,411 |
| S23 | AB valid* OR TI valid* | 998,470 |
| S22 | S21 OR ( (MH "Glasgow Coma Scale") OR (MH "Neurologic Examination") | 85,517 |
| S21 | S18 OR S19 OR S20 | 54,518 |
| S20 | AB (neuro* N3 assessment) OR TI (neuro* N3 assessment) | 27,349 |
| S19 | AB gcs OR TI gcs | 19,266 |
| S18 | AB “glasgow coma scale” OR TI glasgow coma scale OR AB “glasgow Le?ge” OR TI (glasgow le?ge) | 14,231 |
| S17 | S15 OR S16 | 11,466 |
| S16 | AB “FOUR score” OR TI “FOUR score | 11,447 |
| S15 | AB (Full Outline of UnResponsiveness) OR TI (Full Outline of UnResponsiveness) | 123 |
| S14 | S13 OR ( (MH "Critical Care+") OR (MH "Critical Care Nursing") OR (MH "Critical Care Outcomes") OR (MM "Intensive Care Units+") OR (MH "Life Support Care+") OR (MH “Intermittent Positive-Pressure Ventilation) OR (MH "Respiration, Artificial+") OR (MH "Ventilator Weaning") OR (MH "Ventilation") OR (MH "Pulmonary Ventilation+") OR (MH "Ventilation-Perfusion Ratio") OR (MH "Cheyne-Stokes Respiration") OR (MH "Positive-Pressure Respiration+") OR (MH "Continuous Positive Airway Pressure") OR (MH "Extracorporeal Membrane Oxygenation") OR (MH "Extracorporeal Circulation+") OR (MH "Continuous Renal Replacement Therapy") OR (MH "Intracranial Pressure") OR (MH "Cerebrospinal Fluid Pressure+") OR (MH "Nervous System Physiological Phenomena+") OR (MH "Intracranial Hypertension+") OR (MH "Intracranial Hypotension") OR (MH "Venous Pressure+") OR (MH "Intracranial Hemorrhages+") OR (MH "Intracranial Hemorrhage, Traumatic+") OR (MH "Sinus Thrombosis, Intracranial+") OR (MH "Intracranial Hemorrhage, Hypertensive") OR (MH "Hematoma, Subdural, Intracranial") OR (MH "Central Venous Pressure") OR (MH "High Pressure Neurological Syndrome") OR (MH "Hematoma, Epidural, Cranial") OR (MH "Brain Diseases+") OR (MH "Subarachnoid Hemorrhage+") OR (MH "Cerebrovascular Circulation+") OR (MH "Neurophysiological Monitoring+") OR (MH "Neurophysiology") OR (MH "Hemodynamic Monitoring") OR (MH "Cerebrovascular Circulation+") OR (MH "Cerebral Infarction+") OR (MH "Cerebral Hemorrhage+") OR ((MH "Jugular Veins") AND (MH "Oxygen Saturation")) OR (MH "Catheterization, Central Venous") OR (MH "Central Venous Catheters") ) | 515,183 |
| S13 | S1 OR S2 OR S3 OR S4 OR S5 OR S6 OR S7 OR S8 OR S9 OR S10 OR S11 OR S12 | 479,370 |
| S12 | AB ( brain N2 (oxygen*) OR PbtO2 ) OR TI ( brain N2 (oxygen*) OR PbtO2 ) | 3,569 |
| S11 | AB ( (“jugular venous” N3 saturation) OR sjvO2 ) OR TI ( “jugular venous” N3 (saturation OR sjvO2)) | 497 |
| S10 | AB ( (cerebral perfusion) OR CPP ) OR TI ( (cerebral perfusion) OR CPP ) | 26,568 |
| S9 | AB ( (intracranial pressure) OR ICP ) OR TI ( (intracranial pressure) OR ICP ) | 45,635 |
| S8 | AB ( ECMO OR ECCOR ) OR TI ( ECMO OR ECCOR ) | 11,871 |
| S7 | AB (extracorporeal N3 (membrane OR carbon dioxide OR CO?2 OR oxygenat* OR removal)) ) OR TI ( (extracorporeal) N3 (membrane OR carbon dioxide OR CO?2 OR oxygenat* OR removal)) ) | 18,044 |
| S6 | AB ( ((mechanical* OR artificial* OR invasiv*) N2 ventil*) ) OR TI ( ((mechanical* OR artificial* OR invasiv*) N2 ventil*) ) | 79,273 |
| S5 | AB “endotracheal tube” OR TI “endotracheal tube” | 10,772 |
| S4 | AB intubat* OR TI intubat* | 67,649 |
| S3 | AB ( (critical* N3 (care OR ill* OR unwell OR condition) ) OR TI ( (critical* N3 (care OR ill* OR unwell OR condition) ) | 113,052 |
| S2 | AB ICU OR TI ICU | 86,095 |
| S1 | AB (intensive care) OR TI (intensive care) | 194,096 |
