# Supplementary figures and images for: The Predictive Validity of the Full Outline of UnResponsiveness Score Compared to the Glasgow Coma Scale in the Intensive Care Unit: A Systematic Review
Source: Neurocrit Care. 2024 Nov 5;43(2):645–58. doi: 10.1007/s12028-024-02150-8 (PMC12436514; doi:10.1007/s12028-024-02150-8)

**Supplementary Material 2.**

**
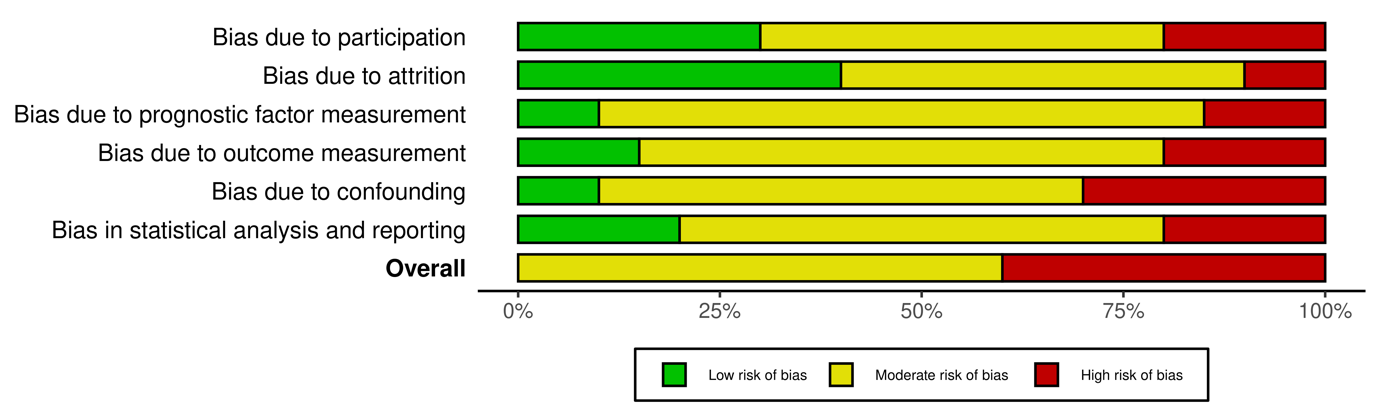
QUIPS Summary Plot**

**
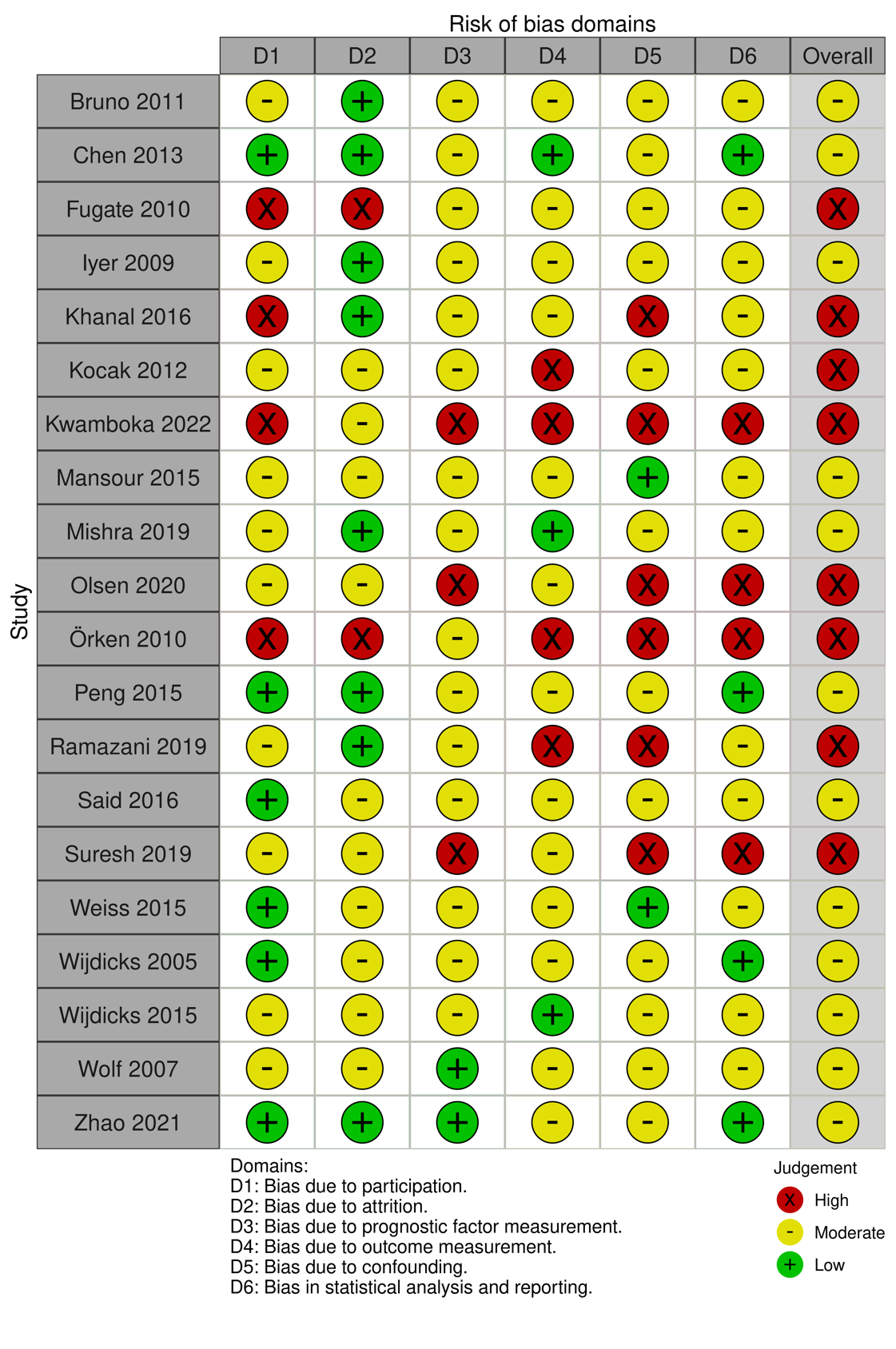
QUIPS Traffic Light Plot**

Supplement: Supplementary file 2 — Supplementary file2 (DOCX 597 kb) [file 12028_2024_2150_MOESM2_ESM.docx]
