# Supplementary material for: The Predictive Validity of the Full Outline of UnResponsiveness Score Compared to the Glasgow Coma Scale in the Intensive Care Unit: A Systematic Review
Source: Neurocrit Care. 2024 Nov 5;43(2):645–58. doi: 10.1007/s12028-024-02150-8 (PMC12436514; doi:10.1007/s12028-024-02150-8)
Supplement: Supplementary file 3 — Supplementary file3 (DOCX 23 kb) [file 12028_2024_2150_MOESM3_ESM.docx]

## Supplementary File 3: ST1. Patient Characteristics

| Study | N | Non-Survivors  n (%) | *Unfavourable* Outcome  n (%) | Age  Mean ± SD | Male  n (%) | Intubated  n (%) | Sedated  n (%) | Principal Diagnosis n (%) | | | |
| --- | --- | --- | --- | --- | --- | --- | --- | --- | --- | --- | --- |
|  |  |  |  |  |  |  |  | TBI | Non-TBI  Neuro. | Post-Arrest/HIE | Other |
| Bruno 2011 | 176 | – | – | 63 ± 15 | 96 (55) | 131 (74) | 0 | 22 (13) | 80 (45) | 33 (19) | 41 (23) |
| Chen 2013 | 101 | 32 (32) | 49 (49) | 64 ± 36.1 | 52 (51) | 91 (91) | 91 (91) | 31 (31) | 70 (69) | – | – |
| Fugate 2010 | 136 | 89 (65) | 101 (74) | 62 ± 15 | 89 (65) | 136 (100) | – | – | – | 136 (100) | – |
| Iyer 2009 | 100 | 33 (33) | 66 (66) | 63.0 ± 18.4 | 55 (55) | 45 (45) | 0 | 3 (3) | 46 (46) | 11 (11) | 40 (40) |
| Khanal 2016 | 97 | 29 (29) | – | – | 50 (52) | – | – | – | – | – | – |
| Kocak 2012 | 100 | 70 (70) | – | 70.5 ± 12.42 | 44 (44) | – | 0 | – | 100 (100) | – | – |
| Kwamboka 2022 | 55 | 36 (65) | – | 41 | 37 (67) | 27 (49) | 20 (36) | – | – | – | – |
| Mansour 2015 | 127 | 25 (20) | 72 (57) | 62.4 ± 1.11 | 59 (47) | – | 0 | – | 127 (100) | – | – |
| Mishra 2019 | 75 | 24 (32) | – | 52.2 ± 14.8 | 32 (43) | – | 0 | – | 75 (100) | – | – |
| Olsen 2020 | 56 | – | – | 63^ (19-86) | 32 (57) | – | – | – | – | – | – |
| Örken 2010 | 124 | 53 (43) | 61 (49) | 68.4 ± 14.7 | 65 (52) | 18 (14) | 0 | – | 120 (97) | – | 4 (3) |
| Peng 2015 | 120 | 26 (22) | 79 (65) | 47.9 ± 14.8 | 85 (71) | 58 (48) | 0 | 53 (44) | 67 (56) | – | – |
| Ramazani 2019 | 300 | 84 (28) | – | 63.36 ± 16.98 | 143 (48) | – | – | – | – | – | – |
| Said 2016 | 86 | – | – | 63^ (50-77) | 51 (59) | 86 (100) | 0 | – | 13 (15) | 17 (20) | 56 (65) |
| Suresh 2019 | 111 | 38 (34) | – | 40.1 ± 17.6 | 80 (72) | 110 (99) | 0 | 51 (46) | 10 (9) | 4 (4) | 46 (41) |
| Weiss 2015 | 85 | 3d 22 (26)  7d 49 (58) | 66 (78) | 60 ± 2 | 63 (74) | 85 (100) | 1d 60 (71)  3d 28 (35) | – | – | 85 (100) | – |
| Wijdicks 2005 | 120 | 25 (21) | 72 (60) | 58.9 | 62 (52) | 57 (48) | 0 | 25 (21) | 81 (68) | 10 (8) | 4 (3) |
| Wijdicks 2015 | 1645 | In-ICU 129 (8)  In-Hosp 191 (12) | – | 60.2 | 836 (51) | 540 (33) | 272 (17) | – | 324 (20) | – | 1421 (86) |
| Wolf 2007 | 80 | 23 (29) | 61 (76) | 64 | 43 (54) | – | 0 | – | 63 (79) | 3 (4) | 14 (18) |
| Zhao 2021 | 271 | In-ICU 26 (10)  In-Hosp 122 (45) | 225 (83) | 56^ | 160 (59) | 72 (27) | 0 | 3 (1) | 228 (84) | 22 (8) | 18 (7) |

*Note. TBI* – Traumatic brain injury; *Non-TBI Neuro.* – neurological illness with non-traumatic aetiology; *Post-Arrest/HIE* – post cardiopulmonary arrest, or hypoxic ischaemic encephalopathy.

^ Expressed as Median (Range) instead of mean ± SD.
