# Supplementary material for: The Predictive Validity of the Full Outline of UnResponsiveness Score Compared to the Glasgow Coma Scale in the Intensive Care Unit: A Systematic Review
Source: Neurocrit Care. 2024 Nov 5;43(2):645–58. doi: 10.1007/s12028-024-02150-8 (PMC12436514; doi:10.1007/s12028-024-02150-8)
Supplement: Supplementary file 4 — Supplementary file4 (DOCX 30 kb) [file 12028_2024_2150_MOESM4_ESM.docx]

**Supplementary File 4. Additional Tables (ST1 to ST6)**

ST1. Comparison of Mean and Median FOUR and GCS Scores

| Study ID | Index Assessment  Timing | FOUR | | GCS | |
| --- | --- | --- | --- | --- | --- |
|  |  | Mean | SD | Mean | SD |
| Chen 2013 | ≤ 24hrs | 5.6 | – | 4.7 | – |
| Khanal 2016 | ≤ 24hrs | 7.89 | 3.87 | 8.27 | 3.82 |
| Mishra 2019 | – | 13.6 | 3.61 | 12.57 | 3.5 |
| Ramazani 2019 | ≤ 24hrs | 8.79 | 4.11 | 8.58 | 3.67 |
| Weiss 2015 | Day 1  Day 3  Day 7 | 2.4  4.3  7.9 | 0.3  0.7  1.1 | 3.5  5.0  6.8 | 0.1  0.5  0.8 |
| Study ID | Index Assessment  Timing | Median | Range | Median | Range |
| Mansour 2015 | 24hrs  72hrs | 11  12 | 9, 15  8, 16 | 8  10 | 7, 11  8, 13 |
| Olsen 2020 | – | 7 | – | 6 | – |
| Said 2016 | ≤ 24hrs | 8.5 | – | 7 | – |
| Zhao 2021 | Adm | 7 | 5, 9ˆ | 5 | 3, 5ˆ |

*Note.* *Adm* – Admission. ˆ IQR.

## ST2. Sensitivity and Specificity of the FOUR and GCS in Predicting Mortality

| Study | Mortality Timepoint | Index Assessment  Timing | Sensitivity (%) | | Specificity (%) | | Cut Off Score | |
| --- | --- | --- | --- | --- | --- | --- | --- | --- |
|  |  |  | FOUR | GCS | FOUR | GCS | FOUR | GCS |
| Fugate 2010 | Discharge | 3 – 5d | 90.50 | 90.50 | 91.1 | 86.7 | 8 | 6 |
| Khanal 2016 | Discharge | ≤ 24 hrs | 79.31 | 75.86 | 79.41 | 77.9 | 6.5 | 6.5 |
| Mansour 2014 | Discharge | 24 hrs  72 hrs | 84.00  100.00 | 84.00  96.00 | 57.00  86.00 | 57.00  92.00 | 11  8 | 8  7 |
| Peng 2015 | Discharge | ≤ 24 hrs | 75.00 | 63.00 | 85.00 | 89.00 | 9 | 7 |
| Ramazani 2019 | Discharge | ≤ 24 hrs | 78.31 | 79.76 | 78.24 | 68.98 | 6.5 | 7.5 |
| Wijdicks 2005 | Discharge | ≤ 24 hrs | 75.00 | 80.00 | 76.00 | 80.00 | 9 | 7 |
| Chen 2013 | 30 days | ≤ 24 hrs | 50.00 | 75.00 | 95.70 | 60.90 | 4 | 5 |
| Mishra 2019 | 28 days | ≤ 24 hrs | 75.00 | 75.00 | 86.27 | 72.55 | – | – |
| Said 2016 | 30 days | ≤ 24 hrs | 84.40 | 84.40 | 79.60 | 77.80 | 9 | 8 |
| Weiss 2015 | 6 months | 1 – 3d | 72.00 | 89.00 | 84.00 | 58.00 | ∆ 3-1d <1 | ∆ 31d <1 |

Note. *∆ 3d-1d <1* – difference between day three and day one scores less than one-point.

Discharge refers to hospital discharge.

## ST3. Comparison of Unadjusted OR for FOUR and GCS Scores in Predicting Hospital Mortality

| Study | FOUR Score | | GCS | |
| --- | --- | --- | --- | --- |
|  | OR | 95% CI | OR | 95% CI |
| Iyer 2009 | 0.75 | 0.68–0.84 | 0.73 | 0.64–0.83 |
| Khanal 2016 | 0.70 | 0.60–0.82 | 0.66 | 0.55–0.79 |
| Mansour 2014 | 0.64^24hr^  0.26^72hr^ | 0.52–0.79  0.12–0.54 | 0.61  0.25 | 0.48–0.77  0.13–0.48 |
| Wijdicks 2005 | 0.80  0.80  0.79 | 0.72–0.88  0.68–0.93  0.68–0.93 | 0.74  0.73  0.72 | 0.65–0.85  0.58–0.90  0.57–0.90 |
| Wijdicks 2015^a^ | 2.72^a,i^  2.76^a,h^ | – | 2.00^a,i^  2.04^a,i^ | – |
| Wolf 2007 | 0.62 | 0.51–0.75 | 0.45 | 0.31–0.66 |

*Note.* ORs are unadjusted and calculated for a one-point increase in FOUR/GCS sum score.

^a^ Odds are calculated for a four-point decrease in FOUR-S and a three-point decrease in GCS-S.

^24hr, 72hr^ Timing of index measure assessment.

^I^ in-ICU mortality. ^h^ in-hospital mortality.

ST4. Frequencies of Lowest Index Measure Scores (FOUR 0 and GCS 3)

| Study | FOUR = 0 | | GCS = 3 | |
| --- | --- | --- | --- | --- |
|  | n | % | n | % |
| Bruno 2011 | 6^a^ | 3^a^ | 15 | 9 |
| Chen 2013 | 5 | 5 | 45 | 45 |
| Fugate 2010 | 35^b^ | 31^b^ | 55 | 49 |
| Iyer 2009 | 9 | 9 | 21 | 21 |
| Örken 2010 | 2 | 2 | 9 | 7 |
| Olsen 2020 | 7 | 13 | 36 | 64 |
| Weiss 2015 | 1d 26  3d 17 | 31  27 | 1d 69  3d 44 | 81  70 |
| Wijdicks 2005 | 9 | 8 | 34 | 28 |
| Wijdicks 2015 | 64 | 4 | 107 | 7 |
| Wolf 2007 | 5 | 6 | 14 | 18 |

*Note.* Xd – index measure timing in days from admission.

^a^ Includes n (%) for FOUR-0 and FOUR-1. b includes n (%) for FOUR-0 – 3.

ST5. Comparison of Lowest FOUR and GCS Scores' Frequencies for Non-Survivors

| Study | Index  Assessment Timing | Overall | | | | Non-Survivor | | | |
| --- | --- | --- | --- | --- | --- | --- | --- | --- | --- |
|  |  | FOUR 0 | | GCS 3 | | FOUR 0 | | GCS 3 | |
|  |  | n | % | n | % | n | % | n | % |
| Bruno 2011 | ≤ 24hrs | 6^a^ | 3^a^ | 15 | 9 | 6^a^ | 100 | 12 | 80 |
| Fugate 2010 | 1-2d | 35^b^ | 31 | 55 | 49 | 35 | 100  100 | 51 | 93 |
| Iyer 2009 | – | 9 | 9 | 21 | 21 | 8 | 89 | 15 | 71 |
| Wolf 2007 | ≤ 24hrs | 5 | 6 | 14 | 18 | 5 | 100 | 13 | 93 |

*Note*. Xd – index measure timing in days from admission.

^a^ Lowest FOUR includes n (%) for FOUR= 0-1.

^b^ Lowest FOUR includes n (%) for FOUR= 0-3.

## ST6. Comparison of Unadjusted OR for FOUR and GCS Scores in Predicting ‘Unfavourable’ Outcome

| Study | FOM Tool & Timepoint | FOUR Score | | GCS | |
| --- | --- | --- | --- | --- | --- |
|  |  | OR | 95% CI | OR | 95% CI |
| Bruno 2011 | GOS 1-3  3m | 0.83 | 0.74–0.93 | 0.81 | 0.71–0.93 |
| Iyer 2009 | mRS 3-6  3m | 0.82 | 0.74–0.93 | 0.82 | 0.74–0.91 |
| Mansour 2014 | mRS 3-6  3m | 0.55^24hr^  0.53^72hr^ | 0.45–0.67^24hr^  0.43–0.65^72hr^ | 0.45^24hr^  0.42^72hr^ | 0.34–0.59^24hr^  0.31–0.56^72hr^ |
| Wijdicks 2005 | mRS 3-6  3m | 0.84 | 0.77–0.92 | 0.83 | 0.76–0.92 |
| Wolf 2007 | mRS 3-6  30d | 0.58 | 0.41–0.82 | 0.67 | 0.54–0.83 |

*Note.* ORs are calculated for a one-point increase in FOUR/GCS sum score.

^24/72hr^ Index measure assessment timing (for studies with multiple assessment episode
